# Drug resistance mechanisms of immunotherapy and translational strategies for reversing resistance for hepatocellular carcinoma: from bench to bedside

**DOI:** 10.3389/fimmu.2026.1808109

**Published:** 2026-04-15

**Authors:** Chenfang Zhang, Dan Zhang, Xin Wen, Shui Liu

**Affiliations:** 1 Department of Hepatobiliary and Pancreatic Surgery, The Second Hospital of Jilin University, Changchun, China; 2 Department of Clinical Laboratory, The Second Hospital of Jilin University, Jilin University, Changchun, China

**Keywords:** drug resistance, hepatocellular carcinoma, immunotherapy, mechanisms, therapeutic strategies

## Abstract

Hepatocellular carcinoma (HCC) is the most common primary malignancy of the liver and remains a major therapeutic challenge. In recent years, immune checkpoint inhibitors (ICIs), represented by PD-1/PD-L1 and CTLA-4 inhibitors, have revolutionized the field of HCC treatment and become the cornerstone of standard immunotherapy regimens, drastically altering the treatment landscape for advanced and unresectable HCC. However, primary and/or acquired drug resistance remains the leading cause of treatment failure, severely limiting the long-term clinical benefits of immunotherapy for HCC patients. This review aims to systematically summarize current research on immunotherapy for HCC, with a focus on drug resistance mechanisms across multiple dimensions: immunosuppressive tumor microenvironment, intrinsic tumor cell factors, and systemic and environmental influences. Potential strategies to overcome resistance are discussed, such as rational combination therapy with antiangiogenic or targeted agents, novel immune targets, nanotechnology-enabled precise delivery, and tumor organoid models for personalized treatment. Future advancements should focus on precision multimodal combinations, innovative therapeutic platforms, and individualized strategies guided by multi-omics and AI, aiming to overcome efficacy bottlenecks and improve patient survival.

## 1 Introduction

Hepatocellular carcinoma (HCC) is a serious challenge to human health worldwide, with incidence and mortality rates ranking among the highest of cancers (1). According to global cancer statistics, liver cancer is the sixth most common malignancy and the third leading cause of cancer-related death worldwide, with HCC accounting for 75%–90% of cases (2). Patients with early-stage disease can achieve long-term survival through radical curative approaches, such as surgical resection and liver transplantation (3, 4). In contrast, for patients who present at intermediate or advanced stages, for whom surgery is not feasible, treatments include transarterial chemoembolization (5), hepatic artery infusion chemotherapy, and systemic drug therapy (6, 7).

In recent years, systemic treatments, particularly immunotherapy, such as immune checkpoint inhibitors (ICIs) and adoptive cell therapy, have achieved breakthrough progress, significantly prolonging survival in advanced HCC (8, 9). However, the application of immunotherapy for HCC faces huge challenges, with drug resistance being particularly critical (10). Drug resistance can be classified as primary, where tumors do not respond to immunotherapy at the initial treatment stage, and acquired, where an initial response is followed by disease progression through a series of adaptive changes. Existing research indicates that only a subset of patients achieves a meaningful response to ICI treatment, resulting in limited objective response rates (11). Furthermore, many patients who initially respond will eventually develop acquired resistance, leading to disease deterioration and other progression (12, 13). Overcoming both primary and acquired resistance is the key to improving immunotherapy outcomes in HCC. Selecting optimal treatment strategies, including the choice among multiple immunotherapy agents and combination regimens to achieve individualized, precise care, continues to be a major challenge in clinical decision-making. Against this backdrop, this review aims to elucidate the primary molecular mechanisms underlying immunotherapy resistance and explore potential strategies to mitigate it, providing a theoretical basis and clinical reference for comprehensive HCC management.

## 2 Therapeutic mechanisms of immunotherapy regimens for HCC

In recent decades, the emergence of immunotherapy has revolutionized the landscape of HCC treatment, offering a paradigm shift by harnessing the body’s own immune system to combat tumor cells rather than directly targeting the malignancy (14, 15). Unlike conventional therapies that often face bottlenecks such as acquired drug resistance and severe systemic toxicities, immunotherapeutic strategies—encompassing immune checkpoint inhibitors (ICIs), adoptive cell therapy (ACT), tumor vaccines, and oncolytic viruses—exhibit unique advantages of durable antitumor responses and favorable safety profiles in subsets of HCC patients. However, the clinical utility of these approaches is still constrained by issues such as limited objective response rates, primary or acquired resistance, and heterogeneous therapeutic outcomes among patients. A comprehensive understanding of the intricate therapeutic mechanisms underlying various immunotherapy regimens, including how they rewire the immunosuppressive tumor microenvironment (TME), reactivate exhausted immune cells, and overcome tumor immune escape, is therefore not only fundamental to interpreting clinical observations but also critical for guiding the development of more effective combination therapies, identifying reliable predictive biomarkers, and ultimately improving the prognosis of patients with HCC.

### 2.1 Immune checkpoint inhibitors

**Figure 1:**
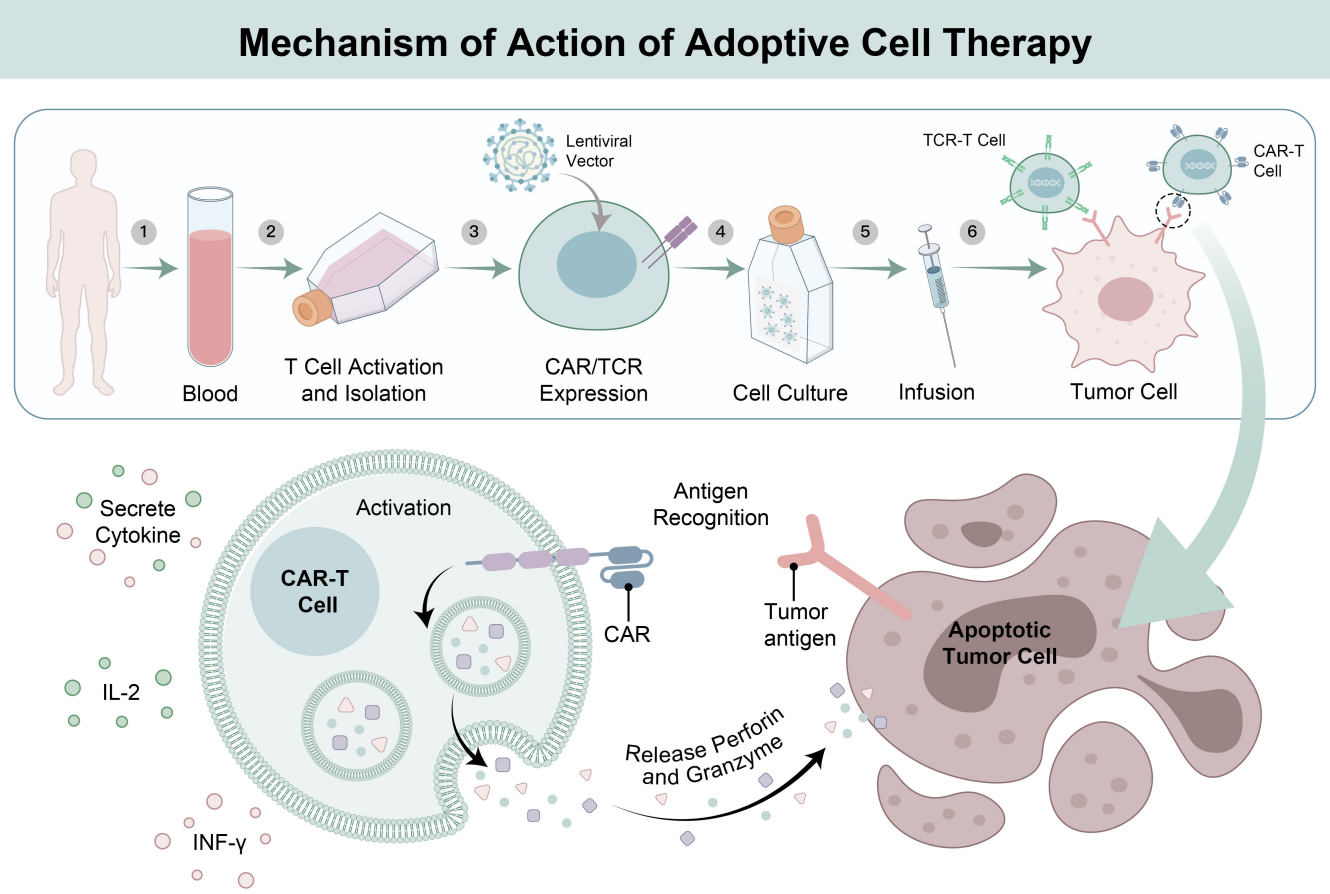
Schematic diagram of the antitumor mechanisms of immune checkpoint inhibitors. Before ICIs treatment, tumor cells highly express immune checkpoint molecules such as PD-L1 and CTLA-4 ligands, which bind to the corresponding PD-1 and CTLA-4 receptors on the surface of T cells, initiating the T cell immunosuppressive signaling pathway. This leads to T cell exhaustion and impaired activation, making T cells unable to effectively recognize and kill tumor cells. Meanwhile, the tumor microenvironment contains suppressive immune cells such as myeloid-derived suppressor cells (MDSCs), regulatory T cells (Tregs) and tumor-associated macrophages (TAMs), which further inhibit the body’s anti-tumor immune response, form an immunosuppressive microenvironment, and enable tumor cells to achieve immune escape and continuous proliferation; ICIs (PD-1/PD-L1 inhibitors, CTLA-4 inhibitors, etc.) block the immunosuppressive signaling pathway by specifically binding to immune checkpoint receptors on the surface of T cells (or ligands on the surface of tumor cells), thereby relieving the immunosuppressive state of T cells and restoring their activation and proliferation capabilities. Activated effector T cells can effectively recognize tumor-specific antigens on the surface of tumor cells and directly kill tumor cells by releasing cytotoxic substances. Meanwhile, activated T cells can recruit effector immune cells such as dendritic cells (DC) and cytotoxic T lymphocytes (CTLs) to infiltrate the tumor microenvironment, form a cascade anti-tumor immune response, eliminate tumor cells.

ICIs block inhibitory receptors (e.g., programmed death 1 [PD-1]) on the surface of T cells in the TME from binding to their corresponding ligands (e.g., programmed death-ligand 1 [PD-L1]) on tumor cells or other cells (16). This removes the inhibitory signals that suppress T-cell activation, reactivating the killing ability of T cells and promoting T-cell proliferation, cytokine secretion, and the recognition and clearance of tumor cells, thereby restoring an effective antitumor immune response (17, 18) (**Figure 1**). Commonly used ICIs include nivolumab, pembrolizumab, atezolizumab, and ipilimumab (**Table 1**). These inhibitors have been proven to significantly improve the objective response rate and overall survival in clinical studies of various solid tumors, such as melanoma (38–40) and urothelial cancer (41).

**Table 1:** Summary of clinical trials of ICIs in HCC.

Target molecule	Immune checkpoint inhibitors	Therapeutic regimen	Clinic trial	Number of patients	Publication year	References
PD-1	Nivolumab	Monotherapy	CheckMate 040	262	2017	(19)
		Monotherapy	CheckMate 459(NCT02576509)	743	2022	(20)
		Monotherapy and in combination with Ipilimumab	NCT03222076	30	2022	(21)
		Combined with Ipilimumab	NCT04039607	668	2025	(22)
	Pembrolizumab	Monotherapy	KEYNOTE-224	104	2018	(23)
		Monotherapy	KEYNOTE-240	413	2020	(24)
		Monotherapy	NCT03062358	453	2023	(25)
		Combined with Lenvatinib	NCT03006926	104	2020	(26)
	Tislelizumab	Monotherapy	RATIONALE-208	249	2023	(27)
		Monotherapy	RATIONALE-301(NCT03412773)	674	2024	(28)
	Sintilimab	Combined with Bevacizumab	NCT03794440	595	2021	(29)
		Monotherapy	ChiCTR2000037655	198	2024	(30)
	Camrelizumab	Combined with Apatinib	NCT03463876	190	2021	(31)
		Combined with Apatinib	NCT04297202	18	2022	(32)
PD-L1	Atezolizumab	Combined with Bevacizumab	IMbrave150(NCT03434379)	501	2021	(33)
	Durvalumab	Combined with Tremelizumab	NCT03298451	1171	2024	(34)
CTLA-4	Ipilimumab	Combined with Nivolumab	NCT01658878	148	2020	(35)
	Tremelimumab	Combined with Durvalumab	NCT02519348	332	2021	(36)
		Combined with Durvalumab	NCT03298451	479	2025	(37)

These inhibitors have encountered numerous challenges in clinical application, including limited response rates and both primary and acquired drug resistance. Addressing these issues requires a deeper understanding of the complex drug resistance mechanisms driven by tumor-intrinsic factors, the immunosuppressive TME and so on (42). To optimize patient selection and maximize benefits, it is also necessary to identify and validate predictive biomarkers (43). Another promising strategy to improve long-term outcomes is the integration of immunotherapy into earlier disease stages. Finally, enhancing therapeutic efficacy through more effective treatment combinations (44), such as pairing ICIs with antiangiogenic drugs (45, 46), or local treatments, has become a core strategy of ongoing clinical research.

#### 2.1.1 PD-1 and PD-L1

The PD-1/PD-L1 signaling pathway is a key route for immune escape by liver cancer cells. PD-1 is a key immune checkpoint protein expressed on the T-cell surface, while PD-L1 is specifically upregulated on malignant tumor cells (47). Within the TME, the persistent presence of tumor antigens chronically activates T cells, causing high surface expression of PD-1, while inflammatory cytokines such as interferon-γ promote PD-L1 overexpression (48). PD-1 binding to PD-L1 initiates downstream immunosuppressive signaling by recruiting Src homology region 2 domain-containing phosphatase-2, which weakens T-cell receptor (TCR) and co-stimulatory signals (49). This cascade inhibits the activation and proliferation of T cells, as well as their release of cytokines (e.g., interleukin-2, interferon-γ, tumor necrosis factor-α), ultimately leading to T-cell exhaustion (50, 51).

PD-1/PD-L1 inhibitors are monoclonal antibodies that bind with high affinity to PD-1 or PD-L1, thereby blocking their interaction (52). This blockade reverses TME-mediated immunosuppression, enabling exhausted T cells to regain their proliferative and cytotoxic functions (53) and restore tumor cell recognition and killing. In 2017, based on data from the multicohort phase 1/2 CheckMate 040 trial, nivolumab received FDA approval as a second-line treatment for liver cancer following sorafenib failure. Subsequently, Thomas Yau et al. conducted a randomized, multicenter, open-label phase 3 trial comparing nivolumab with sorafenib, and the results demonstrated favorable clinical efficacy and safety profiles in patients with advanced hepatocellular carcinoma (HCC) (20).

Despite the clinical benefits of PD-1/PD-L1 blockade monotherapy, its efficacy is often limited by the highly immunosuppressive and angiogenic TME in HCC. Among strategies to overcome these limitations, atezolizumab combined with bevacizumab represents a promising therapeutic approach that integrates immune checkpoint inhibition with anti-angiogenic therapy (54). The VEGF/VEGFR2 pathway is a key mediator of pathological angiogenesis and immunosuppression. It not only drives abnormal tumor angiogenesis but also shapes an immunosuppressive microenvironment by increasing vascular permeability, restricting T-cell infiltration, and inducing the expansion of immunosuppressive cells (55). By inhibiting the VEGF/VEGFR2 signaling pathway, bevacizumab blocks tumor angiogenesis, improves the tumor vascular microenvironment, and promotes T-cell infiltration, thereby enhancing the efficacy of immunotherapy (56). Meanwhile, atezolizumab blocks PD-L1-mediated inhibitory signaling between tumor cells and activated T cells, restores T-cell function, and promotes tumor cell apoptosis. Together, these two agents exert synergistic antitumor effects by remodeling the vascular microenvironment and restoring T-cell function.

#### 2.1.2 Cytotoxic T-lymphocyte-associated antigen 4

Cytotoxic T-lymphocyte-associated antigen 4 (CTLA-4), discovered by Brunet et al. during screening of a cDNA library in 1987, is mainly expressed on the surface of regulatory T cells (Tregs) and activated T cells (57). In 1995, Krummel and Allison from the University of Texas, USA, reported that CTLA-4 negatively regulates immune cells (58), demonstrating that an inhibitory anti-CTLA-4 antibody could cause tumor regression in mouse models.

At the early stage of T-cell activation, CTLA-4, acting as a co-inhibitory receptor, competes with CD28 for binding to CD80/CD86 on antigen-presenting cells resulting in impaired T-cell function (59). CTLA-4 inhibitors (e.g., ipilimumab) block this interaction by binding to CTLA-4 receptors on the surface of T cells, thereby preventing downstream immunosuppressive signaling. This blockade promotes the activation and proliferation of the initial T cells and reduces Treg-mediated immunosuppression.

In addition, ipilimumab combined with nivolumab exerts synergistic effects at different stages of the immune response (60). Ipilimumab mainly acts during the T-cell priming phase in secondary lymphoid organs. By blocking the CTLA-4 pathway, it promotes the activation of naive T cells and the clonal expansion of tumor-reactive T cells, while also alleviating Treg-mediated immunosuppression. Nivolumab mainly acts within the tumor microenvironment. By blocking the PD-1 pathway, it reverses CD8^+^ T-cell exhaustion and restores their function (61). This combination therefore achieves a dual synergistic effect through peripheral immune activation and local immune remodeling (35).

### 2.2 Adoptive cell therapy

#### 2.2.1 Chimeric antigen receptor T-cell therapy

**Figure 2:**
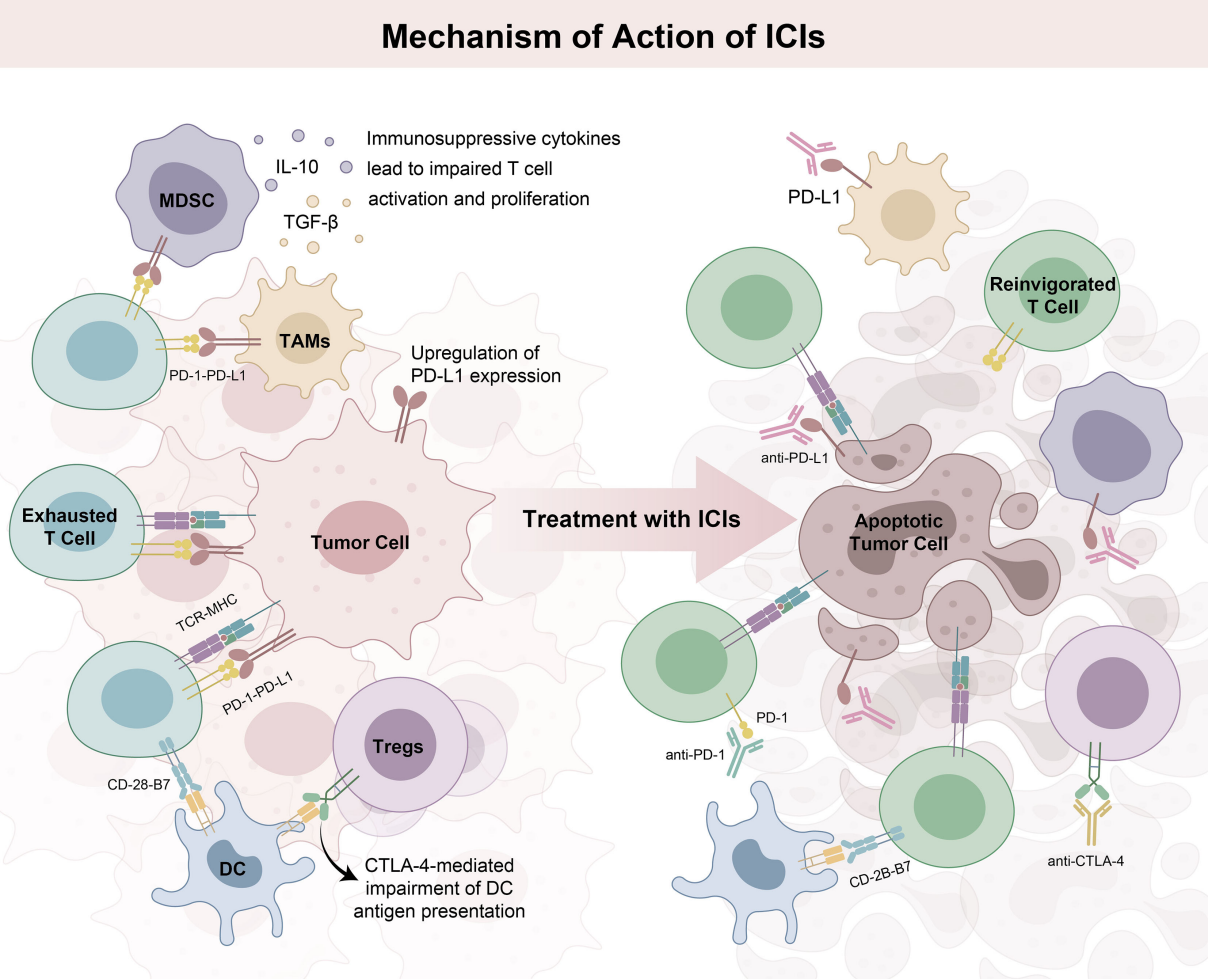
Schematic diagram of the antitumor mechanisms of adoptive cell therapy. The core of ACT is to infuse anti-tumor effector cells (such as CAR-T, TCR-T, NK cells, etc.) that have been expanded, activated and modified (as needed) *in vitro* into patients. After infusion, the effector cells can quickly home to the tumor site, and directly kill tumor cells by secreting cytotoxic substances such as perforin and granzyme through specific recognition of tumor antigens (CAR-T/TCR-T). Meanwhile, they can secrete proinflammatory cytokines to recruit the patient’s own immune cells to infiltrate the tumor microenvironment, synergistically inhibit tumor proliferation, eliminate tumor cell.

Chimeric antigen receptor T-cell (CAR-T) therapy involves genetic engineering a patient’s own T cells to express chimeric antigen receptors (CARs) that recognize tumor-specific antigens (62, 63), enabling a precision killing assault on cancer cells (**Figure 2**). Key target antigens currently under experimental evaluation include mucin 1, glypican-3 (GPC3), alpha-fetoprotein (AFP), natural killer group 2 member D ligands, and hepatocyte growth factor receptor (64). Gao et al. successfully prepared T cells expressing first- and third-generation GPC3-targeted CARs using lentiviral vector transduction (65). In mouse models, these CAR-T cells demonstrated potency against GPC3^+^ HCC cells, highlighting their potential as a therapeutic strategy for GPC3-expressing tumors.

CAR-T cells have achieved remarkable success in the treatment of hematological malignancies and are also being investigated for solid tumors, including liver cancer. However, their application in HCC faces significant obstacles, such as antigen escape, limited CAR-T cell trafficking and infiltration into tumor tissues, the immunosuppressive TME, and CAR-T-related toxicities (62, 64). Compared with single-target CAR-T cell therapy, dual-target CAR-T cell therapy has the advantages of reducing antigen escape of tumor cells, having a higher degree of specificity, and achieving better overall killing effect. In recent years, more and more studies have been devoted to constructing valuable dual-target CAR-T cells (66). A study by Li et al. showed that AFP-GPC3 CAR-T cells are more capable of promoting Th cytokine secretion in Huh-7 cells than AFP CAR-T and GPC3 CAR-T cells, and *in vivo* results show that AFP-GPC3 CAR-T cells are more effective in inhibiting the growth of tumors derived from Huh-7 cells (AFP^+^GPC3^+^) than AFP CAR-T and GPC3 CAR-T cells (67). Even so, addressing these challenges will require further refinement and optimization of therapeutic strategies to enhance their clinical applicability and efficacy.

#### 2.2.2 TCR-engineered T-cell therapy

TCR-engineered T-cell (TCR-T) therapy uses genetic engineering to equip T cells with tumor-specific TCRs (68, 69), enabling precise identification and elimination of cancer cells that present tumor antigens (e.g., AFP or hepatitis B virus [HBV]-derived antigens). Unlike CAR-T, this therapy relies on the patient’s human leukocyte antigen (HLA) genotype for antigen presentation, allowing it to target a wide range of intracellular antigens that traditional drugs cannot reach (**Figure 2**). Meng et al. recruited patients with advanced HBV-related HCC for transplantation with HBV-specific TCR-T cells (70). The results showed that TCR-T therapy is a safe, feasible and potentially effective treatment approach for this patient group. HBV-related HCC cells commonly express HBV antigens, such as HBsAg and HBcAg, and TCR-T cells can precisely target these antigens, enabling specific killing of tumor cells. A study of patients with recurrent HBV-related HCC after liver transplantation by Zhao et al. showed that HBV-specific TCR-T cell therapy had favorable safety and efficacy, and the combination of mRNA-electroporated HBV-TCR-T cells and lentiviral-transduced HBV-TCR-T cells controlled tumor progression and prolonged progression-free survival, with a median PFS of 7.34 months (range, 4.47-7.60 months) (71).

### 2.3 Bispecific antibodies

Bispecific antibodies (BsAbs) are an emerging immunotherapeutic approach that binds simultaneously to target antigens on tumor cells and endogenous effector cells, forming immune synapses, promoting the activation of effector cells, and ultimately inducing tumor cell lysis. The central mechanism of PD-1/PD-L1-CTLA-4 bispecific antibodies is the simultaneous reversal of immune suppression during both the T-cell priming and effector phases, thereby promoting T-cell clonal expansion, restoring CD8^+^ T-cell function, and attenuating regulatory T cell-mediated suppression. Cadonilimab (AK104) is a representative PD-1/CTLA-4 bispecific antibody (72). Wang et al. analyzed data from 78 patients with unresectable HCC and demonstrated that cadonilimab combined with a tyrosine kinase inhibitor had favorable safety and efficacy (73). PD-1/PD-L1-GPC3 bispecific antibodies are engineered to simultaneously recognize and bind two distinct targets (74). Through GPC3-mediated selective binding to hepatocellular carcinoma cells, these agents can promote tumor cell elimination. By additionally blocking the PD-1/PD-L1 pathway, they can restore effector T-cell activity and enhance antitumor immune responses. Li et al. reported that PD-1/GPC3 CAR-T cells exhibited stronger tumor-inhibitory activity than conventional single-target CAR-T cells in a mouse model and prolonged survival (75).

### 2.4 Other immunotherapy methods

#### 2.4.1 Tumor vaccines

Tumor vaccines are designed to exert antitumor effects by enhancing the body’s immune response to tumor-associated antigens. Among them, personalized neoantigen vaccines are based on novel antigens arising from tumor-specific genetic mutations, which are absent in the patient’s normal tissues (76). These antigens, identified through sequencing technologies, are used to produce vaccines with high specificity and strong antitumor immune responses. Other important approaches in HCC vaccine development include dendritic cell vaccines (77) and peptide-based vaccines. Clinical trials have demonstrated that GPC-3 peptide vaccines can be safely administered in advanced HCC and can activate tumor-specific immune responses (78, 79).

#### 2.4.2 Oncolytic viruses

Oncolytic viruses are genetically modified to selectively replicate within and lyse tumor cells (80), without damaging normal tissues (81, 82). In addition to tumor cell lysis, these viruses release tumor-associated antigens and danger-associated signal molecules, which activate antigen-presenting cells such as dendritic cells. This cascade initiates antitumor T-cell immune responses and helps reverse the immunosuppressive state of the TME. Samson et al. conducted *in vitro* and *in vivo* experiments to investigate the oncolytic virus Reo in hepatocytes and animal models (83). Their findings demonstrated that Reo activates the innate immune response, inhibits liver cancer growth, and suppresses replication of oncogenic viruses, suggesting a promising new strategy for treating virus-related liver cancer.

## 3 Classification and mechanisms of immunotherapy resistance in HCC

### 3.1 Classification of immunotherapy resistance

Drug resistance to HCC immunotherapy is a complex process involving multiple factors and steps, encompassing the TME, tumor-intrinsic cellular mechanisms, and systemic and environmental factors. The classification of drug resistance mainly includes primary resistance and acquired resistance (84, 85).

Primary resistance refers to the lack of response to immunotherapy during the initial treatment phase. Its core mechanism lies in the presence of a profoundly immunosuppressive microenvironment before treatment. TME is often enriched with immunosuppressive cells, such as MDSCs and Tregs, which inhibit cytotoxic T-cell activation and promote immune tolerance (86, 87). Another key mechanism involves defects in antigen presentation (88). Tumor cells can downregulate MHC-I or components of the antigen-presenting machinery (APM), leading to failure of CD8^+^ T-cell recognition and thereby depriving immunotherapy of an effective immune-killing foundation from the outset.

Acquired resistance refers to disease progression after an initial response. One of the key factors underlying acquired resistance is clonal evolution of tumor cells, which leads to increased PD-L1 expression and restores inhibitory signaling to T cells (86). In addition, compensatory immune checkpoint pathways, including TIM-3 and LAG-3, may be activated during treatment, promoting T-cell exhaustion and reducing the efficacy of PD-1/PD-L1 inhibitors (86, 89). Furthermore, acquired resistance is often accompanied by metabolic reprogramming of the tumor microenvironment (90). Alterations such as hypoxia and changes in glucose or lipid metabolism can promote Tregs infiltration and suppress immune responses, ultimately leading to disease progression.

### 3.2 Mechanisms of immunotherapy resistance

#### 3.2.1 Immunosuppression of the TME

The TME is a major contributor to immune resistance in HCC (**Table 2**), as these tumors typically exhibit an immunosuppressive state, abundant in various cells and factors that inhibit immune cell function (105–107). Among these components, multiple immunosuppressive cell types interact to jointly construct a barrier that restricts immune cell infiltration and activity (98, 102, 108), with tumor-associated macrophages(CAMs) and cancer-associated fibroblasts (CAFs) playing a central role in driving tumor immune escape through a synergistic regulatory network.

**Table 2:** Immune suppression mechanisms in the tumor microenvironment of HCC and potential therapeutic strategies.

Cell type	Primary mechanism	Potential therapeutic strategy	References
Tumor-Associated Macrophages(TAMs)	1. Promote the immunosuppressive microenvironment through the PKCα/ZFP64/CSF1 axis	1. By blocking the PKCα/ZFP64/CSF1 axis through Gö6976 and lenvatinib	(91)
	2. Inhibit the activity of CD8 T cells through the SRSF10/MYB/glycolysis/lactate axis	2. Pharmacological targeting of SRSF10 with a selective inhibitor 1C8	(92)
	3. Upregulation of sphingolipid synthesis, particularly sphingosine-1-phosphate (S1P)	3. Targeting S1P synthesis with a NEK2 inhibitor or S1P antagonists	(93)
	4. CX3CR1+ TAMs drive T-cell exhaustion via IL-27.	4. Targeting CX3CR1+ TAMs	(94)
Regulatory T cells (Tregs)	1. TGF-β1 induces SOX18, leading to upregulation of PD-L1 and CXCL12.	1. CXCR4 inhibitor or TGFβR1 inhibitor in synergy with anti-PD-L1	(95)
	2. Enrichment of stem-like CCR4 Tregs	2. Targeting intratumoral stem-like CCR4 Tregs	(96)
	3. Loss of E3 ubiquitin ligase *Riplet* modulates fatty acid metabolism to promote terminal exhaustion of CD8 T cells	3. Fatty Acid Synthase (FASN) inhibitor	(97)
Myeloid-derived suppressor cells (MDSCs)	THBS1^+^ Mreg cells mediate immunosuppression via TREM1	Targeting the THBS1/TREM1 axis	(98)
Tumor-associated neutrophils (TANs)	1.Tumor cells reprogrammed CD10ALPL neutrophils to induce the exhaustion of T cells	1. Targeting CD10ALPL neutrophils	(99)
	2. CT10 regulator of kinase-like (CRKL) overexpression nullifies anti-PD-1 treatment efficacy by mobilizing tumor-associated neutrophils (TANs)	2. CRKL inhibitors	(100)
	3. PLAUR neutrophils shape the immunosuppressive microenvironment	3. Blocking PLAUR signaling	(101)
Cancer-associated fibroblasts (CAFs)	1. POSTN CAFs hinder effective T-cell infiltration and decrease the efficacy of immunotherapy.	1. Targeting POSTN	(102)
	2. FMO2+ CAFs enhance the efficacy of anti-PD-1 therapy	2. Activating or reprogramming FMO2+ CAFs	(103)
Tumor-associated endothelial cells (TECs)	CXCL12 TECs impede the differentiation of CD8 naïve T cells into CD8 cytotoxic T cells by secreting CXCL12, and they attract myeloid-derived suppressor cells (MDSCs).	Targeting CXCL12 TECs	(104)

Specifically, SPP1+ TAMs secrete TGF−β to activate the Smad3 pathway in CAFs and IL−6 to activate the STAT3 pathway (109); the crosstalk and synergy between these two pathways promote the transdifferentiation of CAFs into myofibroblast phenotypes and the robust secretion of CCL2 and CCL17, which recruit Tregs into tumors through the CCR4 axis. Tregs themselves highly express CTLA-4, which binds to B7 molecules on the surface of antigen-presenting cells (APCs); collectively, multiple inhibitory signaling pathways (including PD-1/PD-L1, CTLA-4/B7, and TIM-3/Gal-9) act on CD8+ T cells, leading to their functional exhaustion, impaired proliferation, and terminal differentiation. SPP1+ TAMs and CAFs also reinforce each other through a positive feedback loop: CAFs secrete CXCL12 and M−CSF to promote the differentiation of monocytes into M2−like TAMs, while SPP1 secreted by TAMs further enhances CAF activation and extracellular matrix(ECM) deposition, forming a “tumor immune barrier”-like structure that ultimately mediates immunotherapy resistance. Using multiomics approaches, Yao and colleagues characterized the spatial architecture of this barrier, driven by interactions between secreted phosphoprotein 1 (SPP1)^+^ macrophages and cancer-associated fibroblasts within the TME, and found that tumor regions with low CD8^+^ T-cell infiltration exhibited a significantly increased diversity of lipid species, confirming that immunosuppression is associated with lipid accumulation (110). Notably, SREBP1-mediated lipid metabolism promotes the polarization of macrophages toward an immunosuppressive phenotype, thereby hindering CD8^+^ T-cell infiltration (111), suggesting that targeting the SREBP1-regulated lipid metabolic pathway may therefore represent a promising strategy to overcome immunotherapy resistance in HCC.

Other key mediators of TME immunosuppression include IDO/TDO, ARG1/ARG2, and myeloid-derived suppressor cells (MDSCs). In tumors with high IDO/TDO expression, aberrant activation of the AHR pathway enhances Tregs suppressive function and M2-type macrophage polarization, which synergistically suppress the antitumor activity of CD8^+^ T cells. The use of selective AHR inhibitors may therefore remodel the tumor microenvironment and restore T-cell function (112). ARG1/ARG2 contributes to tumor immune evasion by depleting L-arginine in the microenvironment, thereby suppressing T-cell proliferation, activation, and effector function (113), and small-molecule inhibitors targeting ARG may reverse immunosuppression and enhance the efficacy of ICIs. An early study showed that peg-Arg I significantly induced the expansion and accumulation of granulocytic MDSCs through the GCN2 kinase pathway (114), and MDSCs themselves highly express ARG1, further depleting arginine and consequently impairing T-cell function (115). Additionally, accumulation of L-arginine caused by ARG1 inhibition and mTOR activation form a metabolic-signaling synergistic network that drives the occurrence and development of metabolic dysfunction(MAFLD)/non-alcoholic steatohepatitis (NASH)-related HCC. Both can serve as combined biomarkers and therapeutic targets for NASH-related HCC (116).

Beyond these cell types and molecules, Additionally, the TME is rich in tumor-associated neutrophils (99), M2-type macrophages (117, 118), Tregs (119), and immunosuppressive cytokines (95, 104) (e.g., transforming growth factor-β, C-X-C motif chemokine ligand 12). These cells promote immune tolerance through diverse mechanisms, including T-cell exhaustion, PD-L1 upregulation (95, 120), and metabolic reprogramming (93) (e.g., lipid synthesis, lactate accumulation), ultimately weakening the efficacy of ICIs such as anti-PD-1/PD-L1 antibodies (87, 100). Collectively, these cells and molecules form a complex immunosuppressive network that represents a key mechanism underlying immunotherapeutic resistance in HCC.

#### 3.2.2 Intrinsic mechanisms of tumor cells

Tumor cells intrinsically alter their biological characteristics to evade immune surveillance. Key mechanisms include reducing immunogenicity and directly inhibiting the functions of immune cells. Specifically, tumor cells may alter antigen presentation (121–123), upregulate immunosuppressive molecules (e.g., PD-L1) (124), activate internal oncogenic signaling pathways, reprogram cellular metabolism, and acquire stem cell-like characteristics (125) (**Table 3**). For instance, Tong et al. reveals that thePI3K/AKT/mTOR signaling axis, activated by Ubiquitin-conjugating enzyme E2 C(UBE2C)-mediated PTEN ubiquitination and degradation, transcriptionally upregulates Methylenetetrahydrofolate dehydrogenase/cyclohydrolase 2 (MTHFD2) expression, which in turn elevates PD-L1 levels, suppresses CD8^+^ T-cell function, and ultimately promotes tumor immune escape (138). Moreover, the aberrant PI3K/Akt/mTOR signaling pathway can drive IKKβ(the inhibitor of NF-κB) to regulate the inflammatory crosstalk between hepatocytes and immune cells by regulating the NF-κB pathway, thereby promoting the progression of HCC (139). In addition to the PI3K/AKT/mTOR pathway, other signaling axes also contribute to HCC immune escape. A study by Jiang et al. reveals that adaptive upregulation of fibroblast growth factor receptor 1(FGFR1) in HCC cells can drive the recruitment and M2 polarization of TAMs through activation of the MAPK−SPP1 signaling axis, thereby establishing an immunosuppressive TME and conferring resistance to anti−PD−1 therapy (140). Notable, targeted inhibition of FGFR1 can reprogram the TME, restore CD8+ T−cell function, and enhance the efficacy of immune checkpoint blockade therapy in HCC. Another study suggested that HCC develops adaptive resistance to immune checkpoint inhibitors through IFN−γ−dependent upregulation of Indoleamine 2,3-dioxygenase(IDO) 1, providing a potential theoretical basis and therapeutic strategy for overcoming drug resistance in combination with IDO inhibitors (141).

**Table 3:** Intrinsic mechanisms of tumor cells and potential therapeutic strategies.

Classification of resistance mechanisms	Primary mechanisms of action	Potential therapeutic strategies	Reference
Genetic Mutations & Signaling Pathway Activation	1. CTNNB1 mutation leads to reduced CD8+ T cell infiltration	1. Target MMP9 to restore T cell function	(126)
	2. LRP4 mutation promotes anti-PD-1 resistance	2.β-catenin inhibitor	(106)
	3. PPARγ transcriptionally activates increased VEGF-A production, driving MDSC expansion and CD8+ T cell dysfunction	3. Reverse the TME using selective PPARγ antagonist	(127)
Upregulation of Immune Checkpoint Molecules	1. TGF-β1 induces elevated SOX18, upregulating PD-L1 and CXCL12 expression	1.CXCR4 inhibitor or TGFβR1 inhibitor combined with anti-PD-L	(95)
	2. Upregulation of homodimeric PD-1 (Δ42PD-1) expression	2. Anti-Δ42PD-1 antibody	(128)
	3. Expression of the inhibitory receptor TIGIT on lymphocytes and NK cells	3. Dual immune checkpoint blockade (e.g., anti-PD-1 + anti-TIGIT)	(129)
Metabolic Reprogramming & Nutrient Competition	1. Dysregulated lipid metabolism alters the functional states of various immune cells	1. Combine biological therapies targeting lipid metabolism with existing targeted and immunotherapies	(130)
	2. ACVR2A deficiency promotes lactate secretion	2.Lactate dehydrogenase A (LDHA) and monocarboxylate transporter 4 (MCT4) inhibitors	(131)
Epigenetic Regulation	1. H3K36me3-Guided m6A Modification of Oncogenic L1CAM-AS1 Drives Macrophage Polarization and Immunotherapy Resistance	1. Inhibit the L1CAM-AS1-RAN axis	(132)
	2. Lactylation-driven MVP upregulation inhibits PD-L1 degradation	2.Pharmacologically inhibit lactylation	(133)
Resistance to Cell Death	TRIM34 mediates ferroptosis resistance via the UPF1/GPX4 axis	Target TRIM34	(134)
Antigen Presentation Defects	1.Downregulation of MHC class I molecules	1. Targeting PCSK9 in combination with a dual-functional RNA-regulated system	(135)
	2. Pericancerous cross-presentation	2. Redistribute CD103 CTLs	(136)
Cancer Stem Cell Properties	HNRNPM maintains cancer stem cell properties and shapes an immunosuppressive microenvironment	Inhibit HNRNPM	(137)

Beyond oncogenic signaling pathways, metabolic reprogramming also plays a crucial role in tumor immune escape. Under hypoxic conditions in the TME, HIF−1α, as a master regulator of T−cell metabolic adaptation, transcriptionally upregulates key glycolytic effectors including glucose transporters (GLUT1) and glycolytic enzymes (HK2, LDHA), thereby enhancing glycolytic flux and maintaining ATP production. However, the abnormally elevated glycolytic flux mediated by HIF−1α not only leads to excessive intracellular lactate accumulation but also triggers acidosis and imbalance in the TME. These changes further inhibit the phosphorylation of T-cell receptors (TCRs) and block the activation of CD8^+^ T cells, ultimately enabling immune escape and promoting tumor progression (142). Additionally, glutaminase 1 (GLS1)-mediated glutaminolysis provides glutamate for GSH synthesis, which plays a central role in maintaining redox balance in cancer cells and protecting them against ferroptosis (143). Therefore, targeting GLS1 together with GPX4/GPX1 may represent a potential therapeutic strategy for overcoming immune resistance. Li et al. found targeting GLS1 can attenuate the stemness of hepatocellular carcinoma by increasing reactive oxygen species and inhibiting the Wnt/β-catenin pathway in HCC (144). Furthermore, M2 macrophages are closely associated with an immunosuppressive microenvironment and promote tumor proliferation and progression by secreting factors that regulate T cells and angiogenesis; GLS1 can facilitate M2 macrophage polarization and angiogenesis in tumors, thereby contributing to tumor progression (145). Collectively, these mechanisms contribute to the formation of a “cold” or “immune-privileged” TME (146), allowing the tumor to evade T-cell recognition and cytotoxicity, ultimately leading to immunotherapy failure.

#### 3.2.3 Systemic and environmental factors

The gut microbiota profoundly influences the immunotherapeutic response in liver cancer through the gut–liver axis, and its imbalance is an important external contributor to ICI resistance (147). The composition of the intestinal microbial community can regulate immune status both locally in the liver and systemically. For instance, an intestinal environment enriched with beneficial bacteria such as *Akkermansia muciniphila* is associated with improved responses to PD-1 treatment (148, 149), whereas intestinal dysbiosis may disrupt this favorable immune state (150). The gut microbiota and its metabolites, such as bile acids, can enter the circulation and directly or indirectly influence the immune response within the TME by regulating the activity of myeloid-derived suppressor cells and tumor-associated macrophages (14, 147). Similarly, short-chain fatty acids produced by the microbiota affect dendritic cell maturation and T-cell differentiation, shaping the intensity of antitumor immune responses (147, 151). Reduced microbial diversity or loss of beneficial bacterial species, due to antibiotic use or the disease itself, can weaken immunotherapeutic efficacy and even lead to primary resistance (14, 152).

Strategies to restore a healthy intestinal ecosystem, such as fecal microbiota transplantation(FMT), have been explored as potential approaches to overcome HCC immunoresistance and enhance the efficacy of PD-1/PD-L1 inhibitors (153, 154). Baruch et al. demonstrated in a clinical trial that fecal microbiota transplantation (FMT) can overcome resistance to PD-1 immunotherapy in patients with advanced melanoma (155). By remodeling the gut microbiota, FMT can activate intestinal antigen-presenting cells and subsequently enhance the infiltration and effector function of intratumoral CD8^+^ T cells, thereby reversing resistance to immunotherapy. Wu et al. established a MAFLD-HCC mouse model and demonstrated that Akk combined with a PD-1 inhibitor could significantly suppress tumor growth and enhance T-cell infiltration and activation (156). Akk can reduce serum lipopolysaccharide (LPS) levels and bile acid metabolites by repairing the intestinal mucosal barrier, while also decreasing the numbers of m-MDSCs and M2 macrophages (148). In addition, the same group collected and analyzed fecal samples from patients with HCC, showing that enrichment of Akk may enhance the clinical response to PD-1 therapy and prolong survival, suggesting its potential as a biomarker for predicting immunotherapy efficacy.

## 4 Clinical subtype stratification and bench-to-bedside integrative analysis

### 4.1 Clinical subtype stratification based on etiology

#### 4.1.1 HBV-related HCC

HBV-related HCC is characterized by chronic immune tolerance caused by persistent exposure to viral antigens (157). Sustained expression of HBV antigens leads to T-cell exhaustion (158), accompanied by increased co-expression of PD-1 and TIM-3, which reduces the efficacy of immunotherapy (159, 160). Mechanistically, HBx upregulates PD-L1 expression through activation of the NF-κB signaling pathway, thereby suppressing T-cell activation and promoting resistance to ICIs (161). In addition, chronic HBV infection promotes the production of immunosuppressive cytokines and the expansion of Tregs within TME (162, 163). Combining antiviral therapy with immunotherapy may suppress viral replication, reduce chronic antigen stimulation, and improve resistance to immunotherapy (164).

#### 4.1.2 HCV-related HCC

HCV-related HCC is characterized by expansion of MDSCs and increased expression of immunosuppressive cytokines, such as IL-10 and TGF-β (165). The core mechanism of resistance in this subtype is CD8^+^ T-cell exhaustion (166), which results from persistent inflammatory stimulation and metabolic abnormalities during chronic HCV infection. In addition, liver fibrosis associated with chronic HCV infection promotes stromal remodeling and immune resistance (157, 167, 168). Combination therapy with antifibrotic agents and ICIs may enhance immune cell infiltration and improve immunotherapeutic efficacy.

#### 4.1.3 Non-viral HCC

In recent years, the incidence of non-viral HCC(MAFLD/NAFLD/NASH-related) has increased significantly. This subtype is characterized by metabolic dysfunction (169). Lipid accumulation in hepatocytes promotes lipotoxicity and oxidative stress, leading to mitochondrial dysfunction and increased production of ROS (170). Suppression of immune cell function mediated by metabolic reprogramming is the central mechanism of resistance in this subtype (171). Therefore, combining metabolic modulators with immunotherapy may represent a promising strategy to overcome resistance in this setting.

### 4.2 Clinical subtype stratification based on BCLC stage

The mechanisms of immunotherapy resistance also evolve with tumor progression across BCLC stages. In the early stage, immune resistance is mainly attributed to minimal residual disease (MRD) (172). In intermediate and advanced stages, resistance is associated with reduced tumor immunogenicity, abnormal activation of signaling pathways, and changes in TME.

### 4.3 Clinical subtype stratification based on treatment history

Treatment history can also influence the efficacy of immunotherapy in HCC. Recurrent HCC after surgery or locoregional therapy is often associated with alterations in TME and reduced immune cell infiltration (173). In contrast, HCC after targeted therapy often exhibits adaptive activation of multiple kinase signaling pathways, including the MAPK and PI3K/AKT pathways, together with increased PD-L1 expression, resulting in a complex resistance phenotype (174).

## 5 Outlook

**Figure 3:**
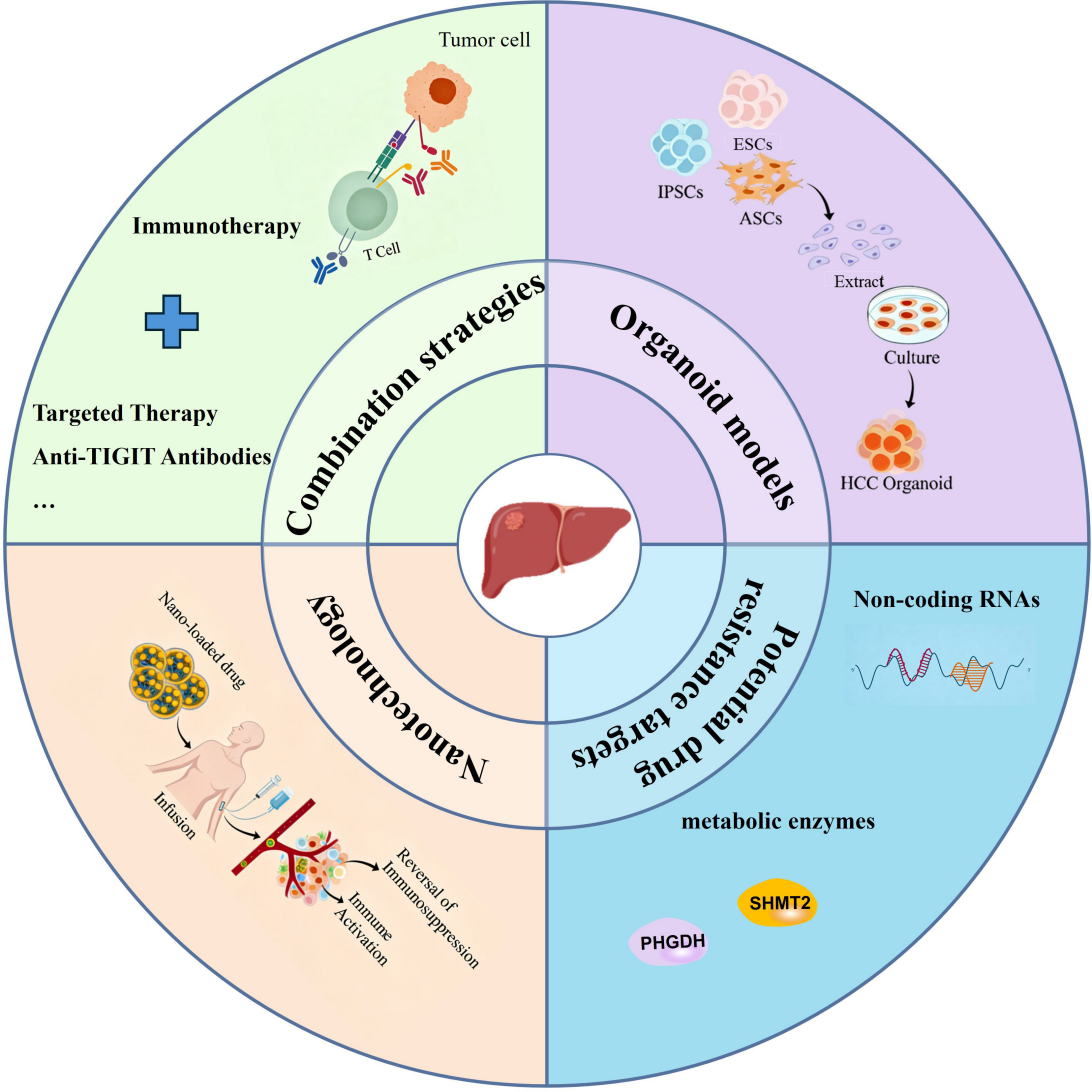
Future perspectives on strategies to improve immunotherapy resistance in hepatocellular carcinoma. This figure summarizes key strategies to overcome immunotherapy resistance in hepatocellular carcinoma (HCC), including combination therapies (ICIs with antiangiogenic agents, TIGIT-targeted drugs), novel targets (ncRNAs, metabolic enzymes), nanotechnology-based drug delivery systems, and organoid models for personalized therapy and mechanistic research, aiming to improve HCC patient outcomes.

The clinical efficacy of immunotherapy in HCC is largely limited by the development of primary or acquired resistance, which has become a major bottleneck in improving patient prognosis. Therefore, exploring effective strategies to overcome immunotherapy resistance and optimize treatment outcomes is of great clinical significance and scientific value (**Figure 3**).

### 5.1 Combination strategies

To overcome immune resistance in HCC, combination strategies with other therapeutic modalities have become the major focus. Immunotherapy combined with antiangiogenic drugs or multikinase inhibitors, including tyrosine kinase inhibitors, has emerged as the first-line standard treatment for advanced HCC (44, 173, 175), significantly improving patient outcomes. Approaches such as atezolizumab plus bevacizumab therapy (33, 54) and regimens combining carfilzomib with apatinib (31, 176, 177) offer superior survival benefits compared with traditional monotherapy with targeted drugs (178, 179). These strategies achieve therapeutic effects by leveraging antiangiogenic drugs to remodeling the TME, promote immune cell infiltration, and create synergy with ICIs (180).

Anti-T-cell immunoreceptor with Ig and ITIM domains (TIGIT)-based combination therapy also offers a promising new option for patients with ICI-resistant disease. In the phase II LIVERTi trial (129), domvanalimab, an anti-TIGIT antibody, combined with zimberelimab, an anti-PD-1 antibody, demonstrated clear antitumor activity in patients with refractory hepatocellular carcinoma, with an objective response rate of 17.2%, a disease control rate of 62.1%, and a median progression-free survival of 4.4 months. However, the ORR did not reach the prespecified statistical threshold, which may be related to the limited sample size, stringent enrollment criteria, and the low proportion of patients with Child-Pugh class B liver function. In a phase III trial evaluating cabozantinib plus atezolizumab in advanced hepatocellular carcinoma (181), this combination significantly improved progression-free survival compared with sorafenib (median PFS, 6.8 vs. 4.2 months), suggesting that a multitarget tyrosine kinase inhibitor combined with immune checkpoint inhibitors can enhance disease control. However, no significant difference in overall survival was observed between the two groups (median OS, 15.4 vs. 15.5 months), which may be attributable to subsequent-line therapies, tumor biological heterogeneity, or the unexpectedly favorable performance of the control arm.

Future efforts to overcome immunotherapy resistance in HCC should focus on shifting from single-agent ICIs to precise multimodal combinations. In parallel, new immunotherapies targeting tumor-associated macrophages(TAMs), tumor associated neutrophils(TANs) (182), and cancer associated fibroblasts (CAFs) (183) should be developed. Furthermore, multi-omics and artificial intelligence technologies should be utilized to identify high-risk populations, ultimately achieving individualized treatment tailored to specific resistance mechanisms (184).

### 5.2 Potential novel drug resistance targets

In the recent years, non-coding RNAs (ncRNAs), mainly comprising long non-coding RNAs (lncRNAs), circular RNAs (circRNAs) and microRNA(miRNA), have been identified as crucial regulators in the initiation and development of HCC. Notably, ncRNAs are extensively involved in modulating tumor immune escape and therapeutic resistance. Owing to their high specificity, easy accessibility, and adjustable expression, ncRNAs have exhibited considerable potential as novel therapeutic targets for overcoming HCC immunotherapy resistance and improving the efficacy of immunotherapy. LncRNAs and circRNAs can act as molecular sponges for miRNAs, thereby relieving the inhibitory effect of miRNA on target mRNAs and upregulating target gene expression (185). For instance, LINC00963 suppresses T-cell infiltration through the miR-92a-3p/LY6E axis, thereby contributing to tumor immune evasion (186). Existing evidence also indicates that LINC00963 exerts epigenetic regulatory effects by binding to zeste homolog 2, a key epigenetic regulator, and suppressing p21 expression (187).

Increasing insights into the metabolic enzymes have also provided a theoretical foundation for developing novel therapeutic strategies to overcome immunotherapy resistance in HCC. Serine hydroxymethyltransferase 2(SHMT2), which serves as a central metabolic enzyme in serine catabolism, can enhances tumor cell adaptation to hypoxia and oxidative stress and promotes immune evasion by suppressing effector T-cell function through metabolic competition. Previous studies have shown that downregulation of SHMT2 can inhibit HCC development, suggesting that selective SHMT2 inhibitors may represent a promising therapeutic strategy (188). Phosphoglycerate dehydrogenase (PHGDH), the rate-limiting enzyme in the serine synthesis pathway, promotes tumor cell proliferation and the formation of an immunosuppressive microenvironment, and its high expression is significantly associated with poor prognosis (189). Experimental studies have demonstrated that NCT-503(a PHGDH inhibitor) can markedly inhibit cell growth and proliferation by suppressing the catalytic activity of PHGDH (190).

Collectively, lncRNAs and circRNAs, as important regulators of HCC immune resistance, exert their effects through ceRNA networks and epigenetic modifications, and their unique regulatory roles make them potential therapeutic targets for reversing ICI resistance. Combined with metabolic targets such as SHMT2 and PHGDH, targeting ncRNAs may provide a novel combined therapeutic strategy to reprogram the tumor immune microenvironment, overcome immune resistance, and improve the clinical outcome of HCC patients.

### 5.3 Nanotechnology

Emerging nanotechnology and drug delivery systems hold great promise (191, 192), enabling precise and coordinated therapy through intelligent nanocarrier design (193). For instance, nanocarriers co-loaded with ICIs and TME-regulating agents can achieve selective accumulation at the tumor site via targeting ligand modification (194), thereby reversing immunosuppression while activating antitumor immunity. Additionally, nanoplatforms can protect immunomodulatory nucleic acids (e.g., small interfering RNA and mRNA) from degradation and efficiently deliver them into specific immune or tumor cells (194). This approach enables precision knockdown of key drug resistance genes or expression of immunostimulatory factors, addressing drug resistance mechanisms such as T-cell exhaustion and myeloid-derived suppressor cell aggregation. Even more promising are environmentally responsive nanomaterials that control the release of drugs in response to TME conditions (e.g., weak acidity, high reactive oxygen species levels) and incorporate imaging functions, allowing integrated diagnosis and treatment (195). Overall, nanotechnology offers multimechanistic, coordinated intervention by enhancing drug targeting and reducing systemic toxicity, providing a powerful tool for systematically overcoming immunotherapy resistance in HCC.

### 5.4 Organoid models

Organoid models also play an increasingly important role in HCC research and therapy (196), closely mimicking the TME and intratumoral heterogeneity through three-dimensional *in vitro* culture systems (197–200). These models enable high-throughput drug screening (201, 202), personalized treatment strategizing (203), evaluation of immunotherapeutic efficacy (204, 205) (including ICIs (203) and CAR-T cell therapy) and investigation of disease mechanisms. By retaining the genetic and phenotypic characteristics of the primary tumor, organoids overcome many limitations of traditional models. However, challenges such as standardization and lack of vascularization remain. Advances in engineering (206, 207) are expected to address these issues, driving progress toward precision and regenerative medicine in HCC.

## 6 Summary

Immunotherapeutic drug resistance is a highly complex challenge, driven by dynamic interactions among the TME, tumor cells, and systemic factors (87, 88, 105, 208, 209). Despite these obstacles, ongoing elucidation of resistance mechanisms is steadily translating reversal strategies from basic research into clinical practice. Through coordinated efforts across disciplines and levels, comprehensive exploration of all aspects of HCC immunotherapy resistance is expected to overcome efficacy bottlenecks and ultimately deliver longer and better survival outcomes for patients.

## Glossary

ACT: Adoptive cell therapy
AFP: Alpha-fetoprotein
CAFs: Cancer associated fibroblasts
CAR-T: Chimeric antigen receptor T-cell therapy
CARs: Chimeric antigen receptors
CTLs: Cytotoxic T lymphocytes
CTLA-4: Cytotoxic T-lymphocyte-associated antigen 4
GPC3: Glypican-3
HBV: Hepatitis B virus
HCC: Hepatocellular carcinoma
HLA: Human leukocyte antigen
ICIs: Immune checkpoint inhibitors
MDSC: Myeloid-derived suppressor cells
PD-1: Programmed death 1
PD-L1: Programmed death-ligand 1
TAMs: Tumor-associated macrophages
TANs: Tumor associated neutrophils
TCR: T-cell receptor
TCR-T: TCR-engineered T-cell therapy
TME: Tumor microenvironment
Tregs: Tregs regulatory T cells.
